# Retraction Note: Functional screen analysis reveals miR-3142 as central regulator in chemoresistance and proliferation through activation of the PTEN-AKT pathway in CML

**DOI:** 10.1038/s41419-020-2319-1

**Published:** 2020-02-12

**Authors:** Lifen Zhao, Yujia Shan, Bing Liu, Yang Li, Li Jia

**Affiliations:** College of Laboratory Medicine, Dalian Medical University, Dalian, 116044 Liaoning Province China

**Retraction to: Cell Death and Disease**

10.1038/cddis.2017.223 published online 25 May 2017

The Editors-in-Chief have retracted this article because the PTEN-shRNA panel in Fig. 6d appears to be the same as the Anti3142+PTEN-shRNA panel in Fig. 6h. The Editors-in-Chief therefore no longer have confidence in the integrity of the data in Fig. 6. All of the authors disagree with this retraction.

